# Title name-vanishing stent phenomenon on optical coherence tomography in a mycotic coronary aneurysm: a case report

**DOI:** 10.1093/ehjcr/ytaf163

**Published:** 2025-04-05

**Authors:** Jamal Yusuf, Ankur Gautam

**Affiliations:** Department of Cardiology, GB Pant Hospital, Govind Ballabh Pant Institute of Postgraduate Medical Education and Research, 1, Jawaharlal Nehru Marg, 64 Khamba, Raj Ghat, New Delhi 110002, India; Department of Cardiology, GB Pant Hospital, Govind Ballabh Pant Institute of Postgraduate Medical Education and Research, 1, Jawaharlal Nehru Marg, 64 Khamba, Raj Ghat, New Delhi 110002, India

**Keywords:** Vanishing stent phenomenon, Optical coherence tomography, Mycotic aneurysm, Coronary artery pseudoaneurysm, Case report

## Abstract

**Background:**

Coronary artery pseudoaneurysm (PSA) is a rare but serious complication following drug-eluting stent implantation. Mycotic aneurysms can develop from coronary stent infections, which, while uncommon, carry high mortality rates. Early diagnosis and intervention are essential for improving outcomes in such cases. This report presents a case of mycotic aneurysm in the left circumflex artery (LCx) following percutaneous coronary intervention (PCI), with the ‘vanishing stent phenomenon’ observed on optical coherence tomography (OCT).

**Case summary:**

A 62-year-old male was admitted with fever and chest pain 1 week after PCI of the LCx and left anterior descending artery using drug-eluting stents. Despite empirical antibiotic treatment, his fever and chest pain persisted, prompting further investigations. Positron emission tomography-computed tomography (PET-CT) scan revealed significant uptake of fluoro-deoxy-glucose around the LCx stent, suggestive of infection. Coronary angiography revealed a coronary PSA at the LCx stent site, with OCT showing damaged, disappeared and displaced stent struts, known as the ‘vanishing stent phenomenon.’ The patient was diagnosed with stent infection and mycotic aneurysm. He was treated with intravenous antibiotics, followed by successful surgical removal of the infected stent, repair of the PSA, and coronary artery bypass grafting. The patient’s post-operative course was uneventful and he was discharged in stable condition.

**Discussion:**

This case highlights the importance of timely diagnosis and management in coronary stent infections. The ‘vanishing stent phenomenon’ on OCT demonstrated severe infection-related stent damage. PET-CT, OCT imaging, and prompt surgical with antibiotic interventions were crucial in treating this life-threatening complication, emphasizing vigilance after PCI for persistent symptoms.

**Learning points**

- Persistent fever post-percutaneous coronary intervention (PCI) should prompt suspicion of stent infection and immediate investigations.
- Positron emission tomography (PET)-CT should be prioritized if the patient develops unexplained fever, signs of infection, or recurrent systemic symptoms suggestive of ongoing inflammation, especially when traditional imaging is inconclusive.
- Optical coherence tomography (OCT) provides high-resolution imaging to detect biofilm formation, endothelial disruption, stent mal-apposition, and vessel wall damage, enabling early diagnosis and optimizing treatment strategies.
- Combining antibiotics with surgery (stent removal and bypass) is vital for treating mycotic aneurysms.

## Introduction

Coronary artery pseudoaneurysm (PSA) is a rare and often underdiagnosed complication that can occur following the implantation of drug-eluting stents.^1^ A coronary artery aneurysm is defined as a focal dilatation of the artery that exceeds 1.5-times its normal diameter.^2^ Coronary stent infections, though rare, are a devastating complication following percutaneous coronary intervention (PCI). Despite adequate treatment, mortality rates remain high, ranging from 40% to 60%.^3^ Therefore, timely diagnosis and prompt intervention are crucial to enhance patient outcomes. We present a case of mycotic aneurysm formation following PCI of the left circumflex artery (LCx) and demonstrate the ‘vanishing stent phenomenon’ observed on optical coherence tomography (OCT). The condition was successfully treated through a combination of antibiotic therapy and immediate surgical intervention.

## Summary figure

**Table:** 

Day	Event
Day 0	Index procedure: percutaneous coronary intervention (PCI) performed with drug-eluting stents in left circumflex artery (LCx) and left anterior descending (LAD).
Day 3	Patient was discharged in stable condition following the PCI.
Day 7	Patient developed high-grade fever and was re-admitted to the same centre with fever and chest pain.
Day 9	Empirical antibiotic therapy initiated with i.v. ceftriaxone and vancomycin.
Day 15	Patient referred and admitted to our tertiary care centre due to unresolved fever and chest pain.
Day 16	Positron emission tomography (PET)-CT scan revealed significant uptake of fluorodeoxyglucose around the LCx stent, suggestive of infection.
Day 17	Coronary angiography and optical coherence tomography (OCT) imaging identified a mycotic aneurysm at the LCx stent site along with the ‘vanishing stent phenomenon’.
Day 21	Gradual normalization of inflammatory markers; patient became afebrile.
Day 23	Surgical intervention: infected stent removed, coronary pseudoaneurysm (PSA) repaired, and coronary artery bypass grafting performed.
Day 28	Patient was discharged successfully in stable condition after an uneventful recovery.

## Case presentation

A 62-year-old hypertensive, current smoker, and non-diabetic male, was referred to our centre with a 1-week history of fever following PCI, with no identifiable cause. Two weeks earlier, the patient was hospitalized at a peripheral centre for unstable angina, where coronary angiography revealed critical stenosis in the left anterior descending (LAD) and LCx arteries. Subsequently, PCI was performed, with a 3 × 38 mm Zotarolimus-eluting stent (Resolute Onyx, Medtronic, CA, USA) deployed in the LCx and a 2.75 × 33 mm Everolimus-eluting stent (Xience Prime, Abbott Vascular, Santa Clara, CA, USA) placed in the LAD. Pre-dilatation was performed using a balloon smaller than the artery (less than a 1:1 artery-to-balloon ratio), and post-dilatation was carried out with a 1:1 balloon-to-stent diameter ratio at 16 atm pressure. Notably, high-pressure balloon dilatation was not utilized, and the PCI was guided by angiography rather than intravascular imaging. Patient was discharged on guideline directed medical therapy.

One week after the index procedure, the patient was re-admitted to the same centre due to the development of high-grade fever accompanied with chills and persistent chest pain. Initial investigations were conducted to identify the cause of the fever, and simultaneously intravenous antibiotics were initiated with ceftriaxone 1 g i.v. every 12 h and vancomycin 1 g i.v. every 12 h keeping the possibility of stent infection in mind. Despite treatment with 6 days of empirical antibiotics, the patient did not show significant improvement, prompting referral to our higher centre for further evaluation with suspicion of stent site infection.

**Figure 1:**
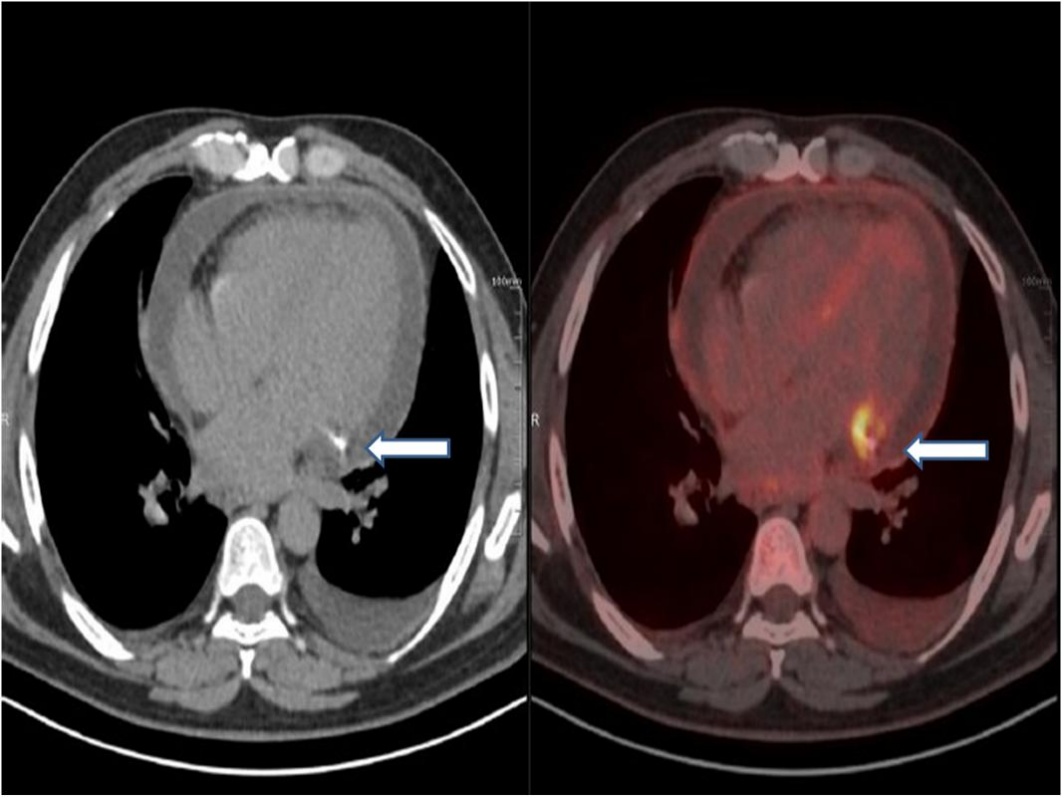
Positron emission tomography-CT imaging revealing elevated FDG activity surrounding the left circumflex artery stent in the left atrioventricular groove (arrow denotes), indicative of inflammation or infection at the stent site.

**Figure 2:**
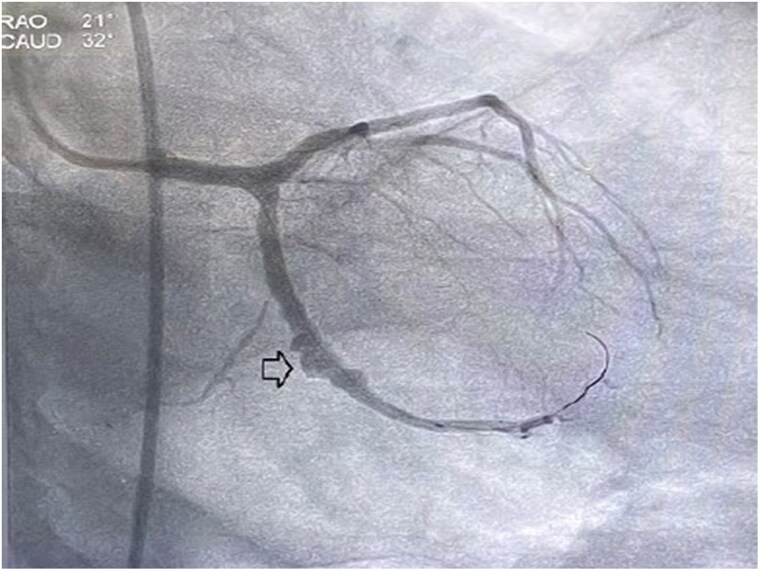
Coronary angiography demonstrating patent stents in the left anterior descending and left circumflex artery, with a coronary pseudoaneurysm identified at the left circumflex artery stent site (arrow denotes), highlighting the presence of vascular abnormalities.

**Figure 3:**
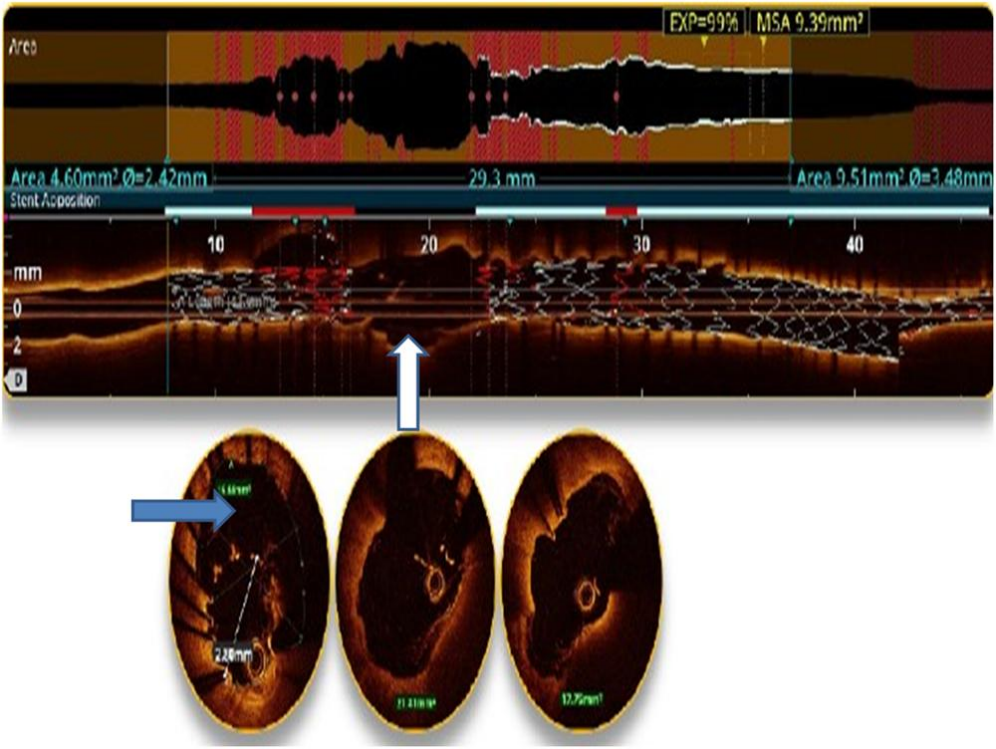
Optical coherence tomography imaging showing displaced and damaged stent struts, with a sizable cavity adjacent to the stent (rightward-facing arrow) and areas of stent disappearance (upward-facing arrow), consistent with stent degradation and structural failure due to infection.

At current admission, the patient continued to experience fever and persistent chest pain. He remained haemodynamically stable, and cardiovascular examination revealed normal findings. Electrocardiography showed no dynamic changes, and echocardiography revealed preserved left ventricular function with no regional wall motion abnormalities, and a mild pericardial effusion was observed. Inflammatory markers were elevated, with a total leucocyte count of 17 200 cells/μL (normal range: 4000–11 000 cells/μL), serum procalcitonin levels of 0.18 ng/mL (normal: <0.05 ng/mL), and C-reactive protein levels of 12.4 mg/dL (normal: <1 mg/dL). Blood cultures, however, were sterile as the patient had already received intravenous antibiotics. A positron emission tomography (PET)-CT scan revealed significant uptake of fluoro-deoxy-glucose (FDG) (SUV max-6.1) around the stent in the left atrioventricular groove, specifically adjacent to the LCx stent (*Figure 1*). Subsequent repeat coronary angiography showed patent stents in both the LAD and LCx arteries but identified a coronary PSA at the site of the stent in the LCx (*Figure 2*). Optical coherence tomography imaging revealed displaced, disappeared, and damaged stent struts, along with a sizable cavity adjacent to the stent, indicative of vessel wall disruption consistent with a PSA in the LCx segment (*Figure 3*) (Supplementary  *Video*). Based on these findings, the diagnosis of stent infection with mycotic aneurysm was confirmed.

**Figure 4:**
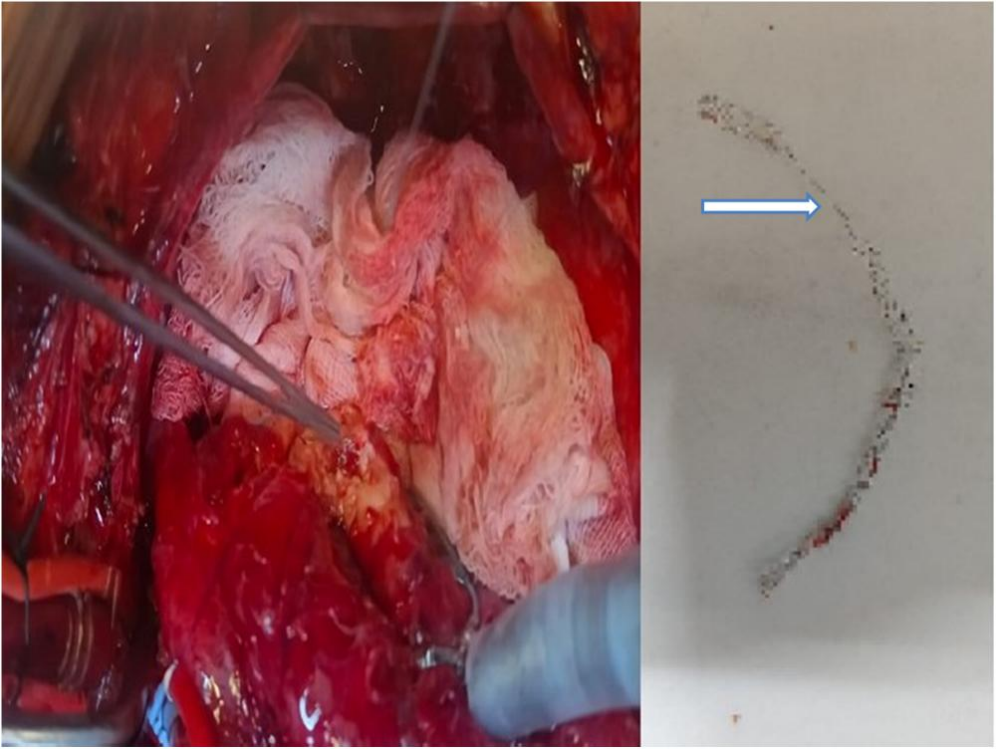
*Left panel:* intraoperative image depicting the surgical repair of a mycotic aneurysm. *Right panel:* surgically removed stent showing significant damage, with structural destruction (arrow denotes) attributed to infection, demonstrating the impact of infectious processes on stent integrity.

The patient remained on intravenous antibiotics, specifically a third generation cephalosporin and vancomycin, with gradual normalization of inflammatory markers and became afebrile in 2 weeks. Subsequently, he underwent surgical intervention. During surgery, the stent was found to be severely crushed and extensively damaged (*Figure 4*), with significant disruption of the vessel wall, likely due to the infective process. Surgical challenges included adhesions and the friable nature of the surrounding vascular tissue, weakened by both infection and inflammation, necessitating careful dissection to prevent further vessel wall damage. The surgeon excised the coronary PSA and proximally ligated the LCx artery. The arterial lumen was inspected for damaged stent fragments, pus pockets, or clots, and the damaged stent was carefully dissected from the arterial wall. To maintain blood flow, a saphenous vein graft bypass was placed distal to the PSA, ensuring continuous myocardial perfusion. This approach effectively treated the PSA while preserving coronary circulation. The extracted stent underwent microbiological and histopathological evaluation, but no microbial agents were detected. This may be due to biofilm formation, which shields bacteria, or prior antibiotic use, which could have suppressed bacterial growth, leading to negative culture results despite strong clinical suspicion of infection. The patient had an uneventful recovery, remained stable during follow-up without any signs of infection, and regained near pre-operative functional capacity within 6 weeks after surgery.

A plan for repeat PET-CT was made if there were any concerns about recurrent infections, and coronary angiography would be considered if ischaemic symptoms developed. Antibiotic therapy followed the standard infective endocarditis protocol (4 weeks).

## Discussion

This is, to our knowledge, the first report of the ‘vanishing stent phenomenon’ observed via OCT in a mycotic coronary PSA. The disappearance of stent struts on OCT, alongside the formation of a mycotic aneurysm, underscores the vital role of advanced imaging in diagnosing and managing rare, life-threatening complications. In this patient, the mycotic aneurysm resulted from stent infection, a rare but potentially fatal complication with high mortality rates, even with appropriate treatment. Coronary stent infections can lead to serious complications such as mycotic coronary PSA, abscess formation, purulent pericarditis, and pericardial empyema.^4^ This case emphasizes the importance of early diagnosis and timely intervention in managing this dangerous condition.

Diagnosing stent-related infections is particularly challenging due to their gradual onset and the potential for systemic symptoms, such as fever and elevated inflammatory markers, which can resemble other conditions.^5^ The diagnostic criteria for coronary stent infections are outlined in *Table 1*.^6^ The delayed diagnosis in our case was due to prior empirical antibiotics and sterile cultures, despite elevated inflammatory markers, leading to misinterpretation as sterile inflammation. To avoid such delays, a high index of suspicion is crucial, even with prior antibiotic use. Blood cultures should be obtained before starting antibiotics, and advanced imaging like PET-CT should be used early for detecting infections when conventional methods are inconclusive. A multidisciplinary approach and close monitoring can ensure timely diagnosis and intervention. Several factors increase the risk of infection, including the reuse of medical devices such as catheters, failure to maintain aseptic technique during procedures, and complications like local infections, haematomas, or PSA. Multiple interventions at the same vascular site and prolonged placement of arterial sheaths further heighten the risk of infection.^7^

**Table 1:** Diagnostic criteria for coronary stent infections

Definitive diagnosis:	The demonstration of an infected coronary artery stent complex by autopsy or surgery
Possible diagnosis:	Any three of the following:
	Placement of a coronary stent in the preceding 3 weeks
	Access site complications or performing multiple procedures via the same arterial sheath
	Bacteraemia
	Fever without any obvious cause
	Leucocytosis
	Acute coronary syndrome
	Imaging (Echo, CT, or MRI) suggestive of inflammation

**Figure 5:**
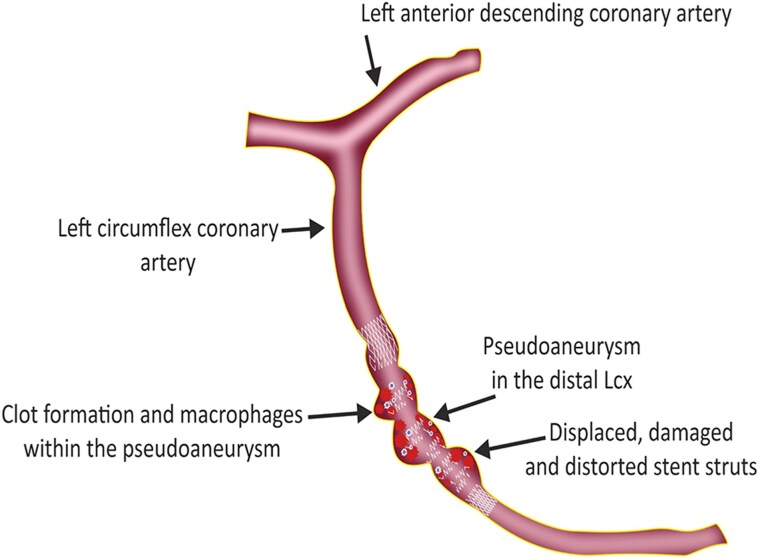
Medical cartoon illustrating the vanishing stent phenomenon in the context of a coronary pseudoaneurysm. The cartoon highlights displaced, damaged, and distorted stent struts (depicted with arrows), as well as inflammatory changes, such as the accumulation of inflammatory cells (shown in the surrounding tissue), explaining the underlying mechanisms of stent degradation, and the formation of the coronary pseudoaneurysm.

First-line imaging modalities include echocardiography and coronary angiography. While echocardiography may detect complications such as coronary aneurysms or pericardial fluid, it may not provide sufficient detail in all cases. Coronary angiography remains the gold standard for identifying coronary PSA.^3^ In this case, both PET-CT and OCT were crucial in confirming the diagnosis. Positron emission tomography (PET)-CT provided valuable insights into the metabolic activity around the stent, indicating infection, while OCT revealed the vanishing stent phenomenon. The ‘vanishing stent phenomenon’ observed in this case refers to the progressive degradation and structural disintegration of the stent, where the stent struts appear damaged, disappeared, and displaced (*Figure 5*). This phenomenon reflects the intricate interaction between infection, stent integrity, and vessel wall weakening. A key mechanism behind this phenomenon is biofilm formation, which likely played a significant role in promoting chronic inflammation and infection around the stent. Biofilm can shield bacteria from immune responses and antimicrobial therapy, contributing to persistent infection and progressive stent degradation. As the biofilm forms, inflammatory cells, and mediators are recruited to the site, exacerbating the damage to the stent and surrounding vessel. Additionally, the ongoing haemodynamic stress and pressure on the vessel wall, coupled with the inflammatory response, can lead to weakening of the vascular structure and loss of the stent’s radial strength. This weakened vessel environment may facilitate the fragmentation and displacement of stent struts, further exacerbating the disintegration process. Ultimately, these combined factors lead to the manifestation of the vanishing stent phenomenon, where the stent appears to lose its structural integrity.^8^

The study by Kumar *et al.*^3^ described a case of stent site infection and PSA formation in the LCx, with PET-CT revealing active FDG uptake around the stent. Their patient underwent coronary artery bypass grafting (CABG) with an uneventful recovery. Similarly, our case also demonstrated focal FDG uptake on PET-CT, and the patient was managed with CABG. However, what sets our case apart is the use of OCT imaging, which uniquely captured the ‘vanishing stent phenomenon’—a striking finding where the stent had started disappearing within the PSA. This rare observation provides critical insights into the structural degradation of infected stents, emphasizing the importance of advanced imaging in understanding coronary stent infections, and their complications.

Currently, there are no established guidelines for managing coronary stent infections or coronary PSA due to the limited number of documented cases. Antibiotic therapy is the cornerstone of treatment. Early stent infections, typically occurring within 10 days of stent placement, tend to respond better to antibiotics. Empirical therapy should cover common pathogens such as *Staphylococcus aureus* and *Pseudomonas*, with a recommended treatment duration of at least 4 weeks.^5^ However, due to the foreign body nature of the stent, removal is often necessary to fully eradicate the infection. Surgical intervention including aneurysm resection, ligation, and distal aortocoronary bypass grafting are often preferred, particularly in cases with a high risk of rupture or delayed coronary complications.^3^ Techniques such as coiling and the use of covered stents are typically avoided due to the risk of bacterial colonization and persistent infection.^9^ In this case, the combination of aggressive antibiotic therapy and surgical intervention was successful. The infected stent was removed, the PSA was repaired, and a CABG surgery was performed. This approach effectively mitigated the risks associated with mycotic aneurysms. The patient’s post-operative recovery was uneventful, highlighting the potential for positive outcomes when timely and appropriate interventions are applied.

## Conclusions

This case underscores the rare but serious complication of mycotic aneurysm formation following PCI, highlighting the critical need for early diagnosis, and intervention in stent-related infections. The vanishing stent phenomenon seen on OCT demonstrated the damaging effects of infection on stent integrity. Despite high mortality rates, timely use of imaging modalities like PET-CT and OCT, combined with prompt surgical intervention and antibiotic therapy, resulted in successful treatment. This case emphasizes the importance of thorough investigations when infection is suspected post-PCI, particularly in patients with persistent symptoms.

## Lead author biography


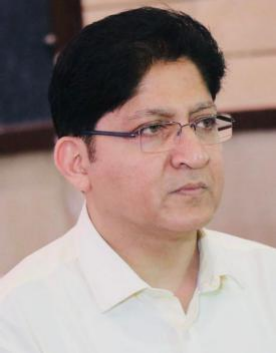


Dr Jamal Yusuf is the Director Professor and Head of the Department of Cardiology at GB Pant Hospital, Delhi. A distinguished academician and dedicated clinician, he has authored over 100 research papers in reputable journals. He is known for his expertise in interventional cardiology and his passion for teaching and mentorship. His commitment to advancing medical knowledge is evident through his active participation in conferences and ongoing research projects. An enthusiastic learner, he continues to explore innovative techniques and approaches in cardiology, making significant contributions to both patient care and the academic community.

## Supplementary Material

ytaf163_Supplementary_Data

## Data Availability

The data underlying this article will be shared on reasonable request to the corresponding author.

## Notes

**Consent:** The patient provided written informed consent for the publication of their case details, including any relevant clinical images, in accordance with ethical guidelines.

**Funding:** None declared.
